# Riga-Fede Disease Associated with Natal Teeth: Two Different Approaches in the Same Case

**DOI:** 10.1155/2015/234961

**Published:** 2015-09-01

**Authors:** Luiz Evaristo Ricci Volpato, Cintia Aparecida Damo Simões, Flávio Simões, Priscila Alves Nespolo, Álvaro Henrique Borges

**Affiliations:** Cuiabá Dental School, The University of Cuiabá, Avenida Manoel Josá de Arruda 3.100, 78065-900 Cuiabá, MT, Brazil

## Abstract

Natal teeth are those present in the oral cavity at the child's birth. These teeth can cause ulcers on the ventral surface of the tongue, lip, and the mother's breast characterizing the Riga-Fede Disease. The treatment depends on the tooth's mobility and the risk of aspiration or swallowing; whether it is supernumerary or regular primary teeth; whether it is causing interference in breastfeeding; breast and oral soft tissue injuries; and the general state of child's health. A 1-month-old female infant was diagnosed with two natal teeth and an ulcerated lesion on the ventral surface of the tongue, leading to the clinical diagnosis of Riga-Fede Disease. The treatment performed consisted of the maintenance of the natal tooth that showed no increased mobility, adding a small increment of glass ionomer cement to its incisal edge, and orientation for hygiene with saline solution. Due to the increased mobility of the other natal tooth, surgical removal was performed. There was regular monitoring of the patient and complete wound healing was observed after 15 days. The proposed treatment was successful and the patient is still in follow-up without recurrence of the lesion after one year.

## 1. Introduction

The eruption of the first primary teeth starts on average around six months old. The presence of teeth at birth or within a month after delivery is rare. These teeth are called natal teeth when they are present at birth and neonatal teeth when they erupted during the neonatal period (first 30 days of life) [1, 2]. The prevalence of natal teeth was estimated to be of 1 : 2000 and neonatal teeth of 1 : 3500 [2]. In most cases these are not supernumerary teeth, but regular primary teeth [3].

Exact etiology of natal and neonatal teeth is not known. Some hypotheses are dominant autosomal inheritance; endocrine disturbance resulting from pituitary, thyroid, and gonads; excessive or increased resorption of overlying bone resulting in early teeth eruption; poor maternal health, endocrine disturbances, febrile episodes during pregnancy, and congenital syphilis [4].

The presence at birth of the lower incisors may lead to the possibility of swallowing and aspiration [4] and the development of Riga-Fede Disease (RFD), which is called the traumatic ulcers that may be located on the ventral surface of the tongue, lip, and the mother's breast [5]. The lesion initiates as an ulcerated area that, with the repetition of trauma, can evolve into an enlarged fibrous mass with ulcerative granuloma appearance. This lesion makes it difficult for the infant to suck and feed, putting the baby at risk of nutritional deficiencies [5].

It is important that professionals are able to recognize the injury and RFD's causal agent so that a proper assessment, diagnosis, and treatment can be performed. Failure to diagnose and properly treat it may result in dehydration and inadequate intake of nutrients by the baby, increasing the potential for infection at the site [6].

This report aims to present the occurrence of Riga-Fede Disease associated with two natal teeth and the treatment approach in a 1-month-old infant.

## 2. Case Report

A 1-month-old female infant attended a private dental office accompanied by her mother for the presence of two teeth since the child's birth. The mother complained that the teeth were making breastfeeding difficult, irritating the infant who could not manage to suck and then cries continuously. The medical history did not reveal any abnormalities and the patient did not use any medication at presentation.

**Figure 1:**
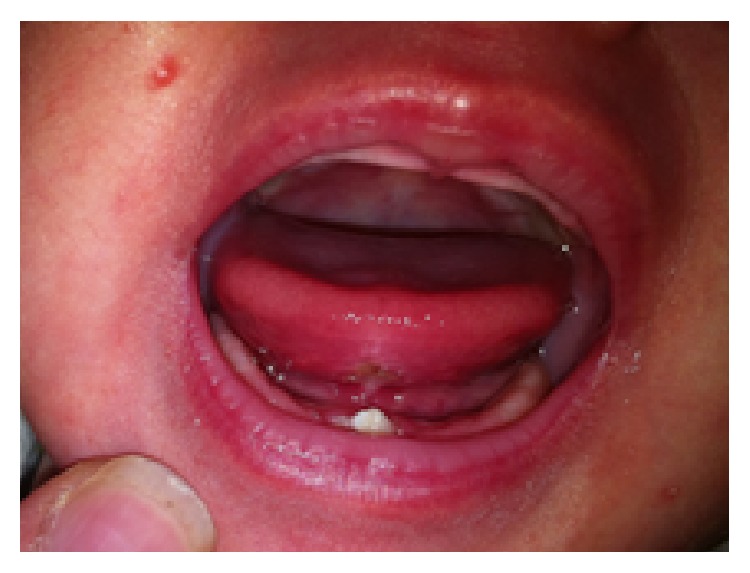
One-month-old infant showing the presence of two natal teeth in the lower arch and ulcerated lesion in the ventral surface of the tongue.

Intraoral examination revealed two natal teeth in the mandibular anterior region and an ulcerated lesion on the ventral surface of the tongue (Figure 1). The lesion had a diameter of 8 mm and was located at the midline anterior portion of the ventral surface of the tongue and seemed to have the impression of the teeth on its center due to repetitive trauma resulting from raking movements of the tongue against the anterior natal teeth leading to the clinical diagnosis of Riga-Fede Disease.

The mother refused permission to perform radiographic examination and tongue biopsy of the child; thus it was not possible at the time to identify if the teeth were supernumerary or regular primary teeth and the histopathological diagnosis of the lesion was not executed.

**Figure 2:**
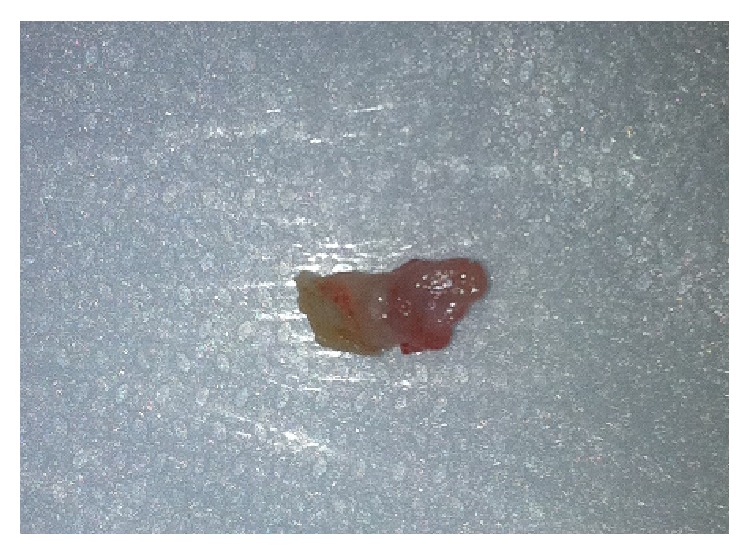
Left side natal tooth surgically removed.

The tooth located on the left side presented grade II mobility, with risk of displacement and consequent swallowing or aspiration, leading to the option of surgical removal (Figure 2). The tooth on the right side with regular mobility was preserved. To prevent the repetition of the trauma on the tongue and to allow wound healing, a small increment of glass ionomer cement was added, covering the cutting edge of that tooth. After carrying out the procedures, the baby was put to breastfeed and the mother reported she was able to suck for a longer period, calming them both. The mother was also instructed to carry out hygiene of the child's tongue wound with saline solution.

**Figure 3:**
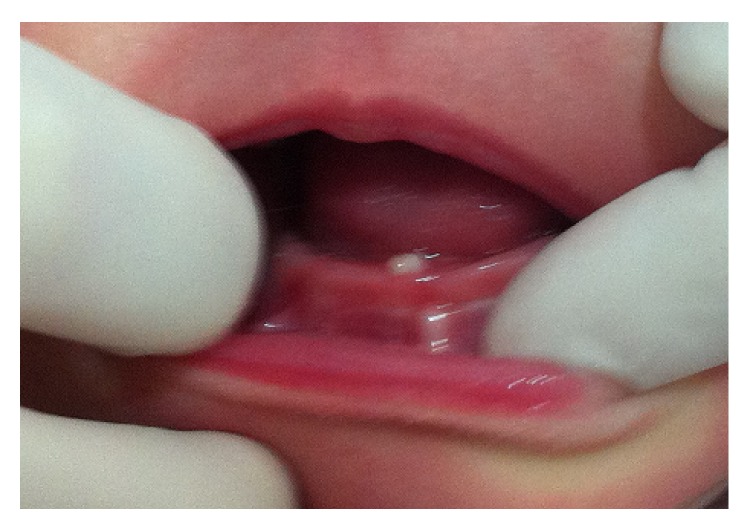
Picture showing complete healing of the lesion 15 days postoperatively.

**Figure 4:**
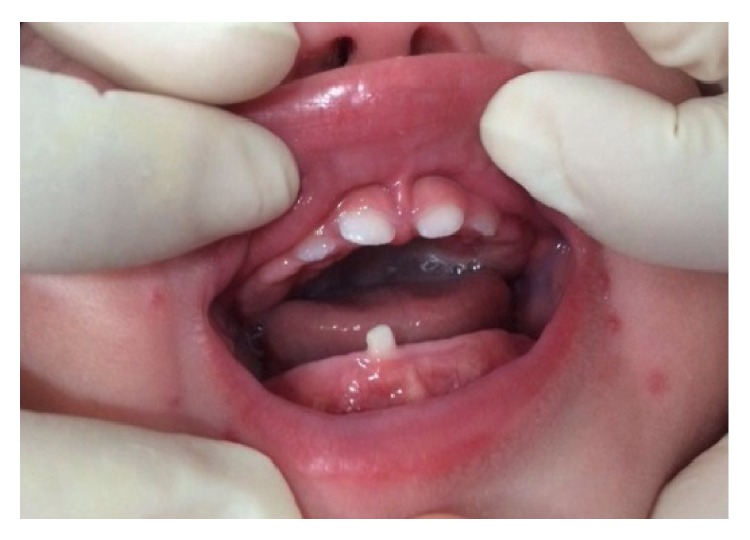
One year of follow-up showing no signs of recurrence of the lesion.

**Figure 5:**
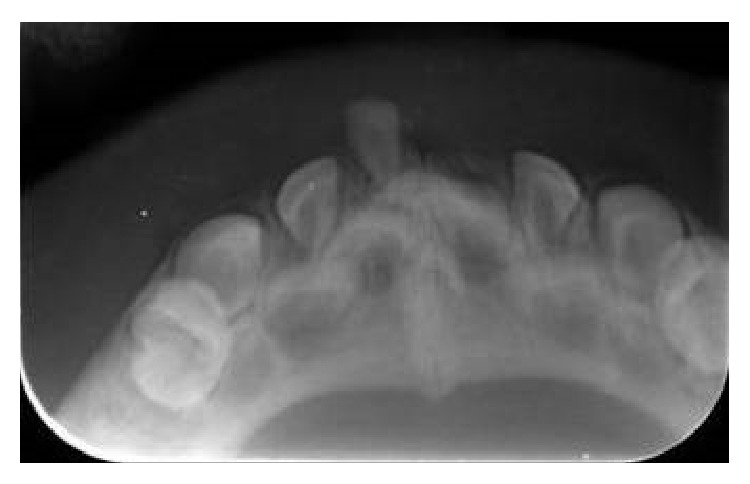
Radiographic aspect of the patient at one year of follow-up showing that the natal teeth were regular primary teeth.

The patient returned after five days showing a good healing of the lesion. After fifteen days, it was completely healed (Figure 3). After one year of follow-up, clinical and radiographic control were performed (Figures 4 and 5). At the time it was confirmed that the natal teeth were regular primary teeth and there were no signs of recurrence of the lesion.

The patient's mother consented to the publication of the child's information along with her photographs and radiographs.

## 3. Discussion

The presence of teeth at birth or within a month after birth is a rare condition. They are classified into two groups according to the time of eruption, as natal teeth, those present at birth, and neonatal, the teeth that erupted in the first 30 days of the child's life [7]. The etiology of the precocious eruption of these teeth is unknown, but the association with many conditions such as infections, nutritional deficiency, fever, endocrine disorders, superficial position of tooth germs, and osteoblastic activity in the area of dental germs has been suggested [5]. The natal and neonatal teeth may be related to heredity and to more than 20 syndromes and abnormalities such as chondroectodermal dysplasia, congenital pachyonychia, Hallermann-Streiff Syndrome, craniofacial dysostosis, Pierre Robin Sequence, Sotos Syndrome, Syndrome of Wiedemann, and Meckel and Gruber Syndrome [2]. In the presented case, the patient had no other congenital abnormalities, nor were there other cases of natal teeth in the family. Her two natal teeth were prematurely erupting primary teeth; however this diagnosis could only be confirmed after one year, with the radiographic examination of the patient.

Although some natal teeth may be supernumeraries, the majority are deciduous teeth that erupted prematurely [1, 8]. Its prevalence is 1 : 2000 live births [2], with preference for females [9]. Possible complications arising from the presence of natal and neonatal teeth include discomfort during breastfeeding, aspiration of teeth, breast nipple abrasions, lingual ulcerations, and refusal to eat [10, 11].

The Riga-Fede Disease corresponds to a lesion on the mucosa of the tongue resulting from trauma by the primary teeth during tongue forward and backward movements. Any surface of the oral mucosa may be affected; however the ventral tongue region is the most common site of occurrence of the ulcer [7]. Failure to diagnose the lesions can lead to deformity or tongue mutilation, dehydration, and inadequate intake of nutrients, resulting in medical sequelae as poor development [12]. In the case reported the patient had irritability and difficulties with breastfeeding because of the injury on the ventral surface of the tongue, which could eventually generate nutritional damage among other problems.

RFD begins as an ulcerated area with prominent raised edges. With repeated trauma, it may progress to an enlarged, fibrous mass with the appearance of an ulcerative granuloma with superficial necrosis. It is histopathologically characterized by an ulcerated mucosa with granulation tissue and a mixed inflammatory infiltrate consisting of lymphocytes, macrophages, mast cells, and an abundant number of eosinophils [13].

Since the patient's mother refused to give permission to perform radiographic examination and tongue biopsy, the diagnosis was based on the complete history and physical examination. van der Meij et al. [13] stated that once the clinician is familiar with RFD, the history and clinical features are most often so typical that there is seldom a need for additional histopathological examination.

The first option for RFD treatment should always be conservative, avoiding natal and neonatal teeth extractions when possible [3]. The removal of the traumatic agent does not mean necessarily that the teeth have to be extracted. Teeth anatomy can be modified by flattening their edges with a finishing bur or by using a polishing disc [6]. The sharp edges can also be covered with adhesive restorations and mouthguards may be used in some cases [7]. If conservative treatment options do not lead to a quick resolution of the injury, the tooth extraction may be needed [6]. Tooth extraction is also indicated when there is excessive tooth mobility because the root is still in initial formation stage, and there is a high risk of swallowing or aspiration, or when they are supernumerary teeth [3, 11, 14, 15]. That was the condition and treatment for one of the teeth. The other one, with regular mobility, was maintained and its cutting edges were covered with glass ionomer cement. The treatment gave immediate comfort to the child who breastfed more calmly and longer and enabled the spontaneous remission on lingual wound.

If the injury persists after extraction of teeth, an excisional biopsy should be performed [6]. In the case described, the lesion healed in fifteen days, requiring no biopsy. After one year the infant had the upper incisors and right side lower central incisor partially erupting. At the radiographic examination it was found that the permanent central incisors were with 1/3 of the mineralized crown.

The premature loss of primary teeth may result in aesthetic, orthodontic, and phonetic problems. It can change the relationship between the jaws and the child could develop harmful tongue posture habits which also jeopardize speech [16]. It can also induce a gingival fibrosis in the area, which could result in difficulties for the eruption of the permanent tooth [17]. Therefore, the close follow-up of the successor permanent tooth eruption is very important [17].

## 4. Conclusion

The presence of natal teeth is rare but can cause the Riga-Fede Disease. Early diagnosis is imperative in order to ensure adequate treatment and prevention of children's malnutrition and dehydration. Removing one of the natal teeth due to its high mobility and the covering of the other tooth's sharp edges proved to be an effective treatment, allowing the resumption of breastfeeding and complete regression of the lesion.
